# Combined N-of-1 trials to investigate mexiletine in non-dystrophic myotonia using a Bayesian approach; study rationale and protocol

**DOI:** 10.1186/s12883-015-0294-4

**Published:** 2015-03-25

**Authors:** Bas C Stunnenberg, Willem Woertman, Joost Raaphorst, Jeffrey M Statland, Robert C Griggs, Janneke Timmermans, Christiaan G Saris, Bas J Schouwenberg, Hans M Groenewoud, Dick F Stegeman, Baziel G M van Engelen, Gea Drost, Gert Jan van der Wilt

**Affiliations:** Department of Neurology, Radboud University Medical Center, PO Box 9101, 6500 HB Nijmegen, The Netherlands; Department of Health Evidence, Radboud University Medical Center, PO Box 9101, 6500 HB Nijmegen, The Netherlands; Department of Neurology, University of Kansas Medical Center, PO Box 2012, KS 66160 Kansas City, USA; Department of Neurology, University of Rochester Medical Center, PO Box 420669, Rochester, NY 14642 USA; Department of Cardiology, Radboud University Medical Center, PO Box 9101, 6500 HB Nijmegen, The Netherlands; Department of Pharmacology-Toxicology, Radboud University Medical Center, PO Box 9101, 6500 HB Nijmegen, The Netherlands; Department of Neurology and Neurosurgery, University Medical Center Groningen, PO Box 30001, 9700 RB Groningen, The Netherlands

**Keywords:** Combined N-of-1 trials, Bayesian, Non-dystrophic myotonia, Mexiletine, Rare diseases, Skeletal muscle channelopathies

## Abstract

**Background:**

To obtain evidence for the clinical and cost-effectiveness of treatments for patients with rare diseases is a challenge. Non-dystrophic myotonia (NDM) is a group of inherited, rare muscle diseases characterized by muscle stiffness. The reimbursement of mexiletine, the expert opinion drug for NDM, has been discontinued in some countries due to a lack of independent randomized controlled trials (RCTs). It remains unclear however, which concessions can be accepted towards the level 1 evidence needed for coverage decisions, in rare diseases. Considering the large number of rare diseases with a lack of treatment evidence, more experience with innovative trial designs is needed. Both NDM and mexiletine are well suited for an N-of-1 trial design. A Bayesian approach allows for the combination of N-of-1 trials, which enables the assessment of outcomes on the patient and group level simultaneously.

**Methods/Design:**

We will combine 30 individual, double-blind, randomized, placebo-controlled N-of-1 trials of mexiletine (600 mg daily) vs. placebo in genetically confirmed NDM patients using hierarchical Bayesian modeling. Our results will be compared and combined with the main results of an international cross-over RCT (mexiletine vs. placebo in NDM) published in 2012 that will be used as an informative prior. Similar criteria of eligibility, treatment regimen, end-points and measurement instruments are employed as used in the international cross-over RCT.

**Discussion:**

The treatment of patients with NDM with mexiletine offers a unique opportunity to compare outcomes and efficiency of novel N-of-1 trial-based designs and conventional approaches in producing evidence of clinical and cost-effectiveness of treatments for patients with rare diseases.

**Trial registration:**

ClinicalTrials.gov Identifier: NCT02045667

## Background

Rare diseases constitute a heterogeneous group of over 6.000 disorders with a prevalence of <1 per 20.000. In Europe, 30 million patients (6 to 8% of the population) have a rare disease [1]. International regulatory authorities such as the Food and Drug Administration (FDA) and European Medical Agency (EMA) accept that it is unreasonable to demand the standard level of evidence (level 1) of multiple Randomized Controlled Trials (RCTs) in building an evidence-base for treatment of rare diseases [2-4]. The ability to conduct RCTs in rare diseases is hampered by low numbers of patients and large clinical heterogeneity. Relying simply on case reports or case series incurs a considerable risk of selection and ascertainment bias. Currently, it is unclear which concessions can be accepted towards the level 1 evidence needed for coverage decisions in case of rare diseases [5-7].

### The case of mexiletine treatment in Non-dystrophic myotonia (NDM)

NDM is a heterogeneous group of monogenetic rare diseases caused by mutations in the skeletal muscle chloride (*CLCN1*) or the sodium ion channel (*SCN4A*) genes. The key symptom is myotonia, a delayed relaxation after voluntary contraction resulting in muscle stiffness [8]. Apart from muscle stiffness, NDM patients also experience functionally limiting complaints of pain, fatigue and weakness [9].

For years, mexiletine (a sodium channel blocker) was considered the drug of choice for NDMs based on clinical experience. The immediate occasion for our study was the decision by the National Health Insurance Board of The Netherlands (and of some other countries), in 2006, to discontinue reimbursement of mexiletine as rational pharmacotherapy for patients with NDM [10,11]. This decision was based on a Cochrane review that reported the absence of two independent level-1 evidence studies showing an effect of mexiletine for NDM [12]. Additionally, because of the lack of precise prevalence numbers of NDM in The Netherlands, the rarity of the disease was taken into question. As a result, many of these patients had to discontinue their mexiletine treatment that seemed clinically effective.

In 2012, the Consortium of Clinical Investigation of Neurological Channelopathies (CINCH) performed an international multicenter cross-over RCT that showed the clinical effectiveness of mexiletine as treatment for patients with NDM over 4 weeks of therapy [13]. Although a prospective RCT is the gold standard, the effort involved in conducting such a trial (as well as time, funding and international cooperation) was substantial, and it will not always be feasible for rare diseases. New innovative trial designs may be used to ameliorate problems with small patient numbers and large clinical heterogeneity.

### Combined N-of-1 trials using a Bayesian approach

In an N-of-1 trial, multiple pairs of active treatment and placebo are offered to an individual patient in a randomized, double-blind fashion, while regularly measuring key endpoints, until efficacy is established or disproved [7].

Due to their design, in which each treatment-pair should be exchangeable in time, N-of-1 trials are especially suitable for the investigation of treatments in chronic, symptomatic conditions, where period effects (i.e. changes in disease state) and carry over effects (i.e. lingering drug effect) are small. This is the case for a number of neurological, reumatological, psychiatric and pulmonary disorders [14]. N-of-1 trials are hardly applicable to surgical, quickly progressive or acute medical conditions [15]. The method has been originally developed to identify the best treatment option for an individual patient in case of genuine doubt concerning treatment benefit by formalization of what a physician does on a daily basis [14,16].

Major advantages of N-of-1 trials from the point of view of the patient and the treating physician are: (1) an N-of-1 trial determines whether the treatment is actually of benefit in the individual patient as opposed to some percentage of a group of patients; (2) the N-of-1 trial avoids the possibly unethical “therapeutic misconception” of the RCT - where most subjects are convinced they are receiving effective treatment even though told there is a 50/50 chance they will receive placebo (and that the treatment may not be effective) [16-19].

More recently, data from multiple N-of-1 trials have been combined (or meta-analyzed) to produce estimates of treatment effect at a population level (i.e. combined N-of-1 trials) [20-22]. For this purpose, Bayesian hierarchical models have been developed [23,24]. Bayesian models use a different approach, which does not rely on the hypothesis testing/confidence intervals paradigm of traditional (frequentist) statistical methods, but allows determination of the posterior probability of whether a treatment effect is beneficial. Additionally, Bayesian methods allow for the use of prior available information (e.g. previous trial results) within the analysis [7].

### Hypothesis

The key hypothesis is that combining data from multiple N-of-1 trials using Bayesian statistics is a promising approach to produce evidence of clinical and cost-effectiveness of treatments for patients with rare diseases. As a case study we will investigate mexiletine in NDM. Results of this approach will be compared with the results of a conventional RCT approach.

Study objectives:

(I). To use Bayesian methods for combining N-of-1 trials to obtain evidence of the clinical and cost-effectiveness of mexiletine in the treatment of NDM, on patient and population level simultaneously.
(II). To compare this approach with a conventional RCT approach.

Both NDM and mexiletine are well suited for an N-of-1 trial design: NDM is a stable disease; mexiletine acts rapidly and its effect quickly subsides upon discontinuation of treatment; effects can be readily and objectively measured; and patients are eager to cooperate.

The Bayesian approach taken in this study will allow for considering questions on patient level and a population level simultaneously and it offers additional flexibility for customizing the trial length for individual patients. Furthermore, there is relevant clinical prior information (data from the previously conducted RCT) that can be incorporated into Bayesian analysis together with our new trial data.

## Methods/Design

### Design

**Figure 1:**
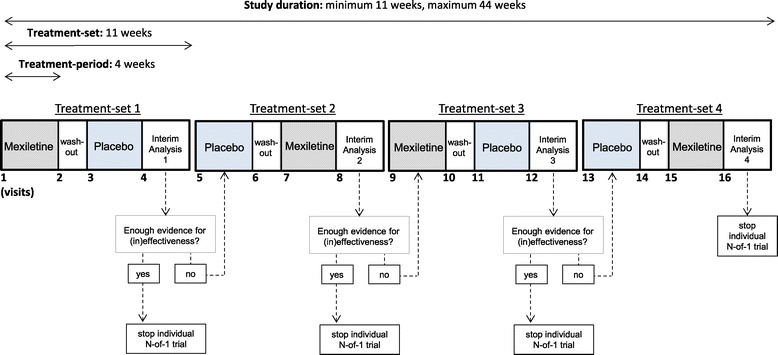
**Study design individual N-of-1 trials.**

In this study a series of double-blind, randomized and placebo-controlled N-of-1- trials is conducted. Each individual N-of-1 trial consists of one to four treatment sets, each comprising 11 weeks. A treatment set comprises two treatment periods: a four-week period of active treatment (mexiletine) and a four-week period of treatment with placebo, with a one-week wash-out in between treatment periods and two weeks for statistical analysis (individual interim analysis) at the end of each treatment set. Within each treatment set, the order in which mexiletine and placebo will be offered will be randomized (block-randomization). Total study duration will be between 11 and 44 weeks per patient, depending on the amount of treatment sets needed to produce convincing evidence of clinical effectiveness or ineffectiveness of mexiletine (Figure 1). Results of the individual N-of-1 trials will be combined to produce estimates of population clinical and cost-effectiveness by using a hierarchical Bayesian model [25]. The trial is performed at the Radboud University Medical Center in Nijmegen, The Netherlands.

### Study population

Based on a sample size calculation (see below), thirty NDM patients will be included. Eligible patients have a genetically confirmed diagnosis of NDM, carry one (or two, in case of autosomal recessive inheritance) causative mutation(s) in the skeletal muscle chloride (*CLCN1*) or sodium (*SCN4A*) channel gene, and are at least 18 years of age and live in The Netherlands, Belgium or Germany.

A previous study in the Dutch NDM population showed a diagnostic yield of 100% for in tandem analysis of these two genes [26]. Inclusion and exclusion criteria, including co-medication are presented in Table 1.

**Table 1:** **Inclusion and exclusion criteria**

**Inclusion criteria**	**Exclusion criteria**
1. At least 18 years of age	1. Inability or willingness to approved to provide informed consent
2. Genetically confirmed diagnosis of NDMs	2. Other neurological conditions that might affect the assessment of the study measurement
	3. Genetically confirmed DM1 (CTG > repeats), or DM2
	4. Existing cardiac conduction defects, evidenced on ECG including but not limited to the following condition: malignant arrhythmia or cardiac conduction disturbance (such as second degree AV block, third degree AV block, or prolonged QT interval >500 ms or QRS duration > 150 msec)
	5. Current use of the following antiarrhythmic medication for a cardiac disorder: flecainide acetate, encainide, disopyramide, procainamide, quinidine, propafenone or mexiletine
	6. Women who are pregnant or lactating
	7. Currently on medication for myotonia such as phenytoin and flecainide acetate within 5 days of enrollment, carbamazepine and mexiletine within 3 days of enrollment, or propafenone, procainamide, disopyramide, quinidine and encainide within 2 days of enrollment
	8. Renal or hepatic disease, heart failure, history of myocardial infarction, or seizure disorders

### Recruitment and screening

Our center of expertise has developed a nation-wide registry of patients with NDM. As described previously, for this registry we asked all Dutch neurologists and the Dutch Patient Association for Neuromuscular Diseases (Spierziekten Nederland), to notify us of patients with NDM. Patients (>18 years) were invited to our neurology outpatient clinic in Nijmegen, The Netherlands, for clinical assessment, needle-EMG, and collection of blood samples for genetic analysis [8,12,26-29]. In 2007, the registry contained 62 NDM patients with a genetically confirmed NMD. Between 2007 and 2014, 48 genetically confirmed patients have been added, resulting in a total of 110 registered patients with extensive phenotypic and genotypic characterization. Asymptomatic carriers are not represented in the registry.

Potentially eligible patients will be selected from the registry. We expect that a maximum of 40% may not be eligible to participate, because of cardiac conditions, kidney or hepatic failure or neurological diseases other than NDM. Out of the remaining patients, random stratified samples will be taken, of 15 patients with a chloride channelopathy (TMC or BMC) and 15 patients with a sodium channelopathy (PMC or SCM). During a screening visit information about the study will be given, and patients will be asked to provide written informed consent. Eligibility will be checked by taking medical history and by conducting venous blood test (blood urea nitrogen, serum chloride, carbon dioxide, creatinine, blood glucose, serum potassium, serum sodium), ECG, and urine pregnancy testing for females. Serum pregnancy testing can be performed if, in the opinion of the investigator, the urine pregnancy test is inconclusive. Dosage, duration and date of last use of phenytoin, carbamazepine or mexiletine will be documented.

We will document characteristics of ineligible or excluded patients to estimate the external validity of our findings. Participants who are taking antiarrhythmics or medication that may affect sodium channels, will have a wash-out period before the baseline visit (Table 1, point 7). For the duration of the study, patients are instructed to comply with the study medication regime as supplied in the treatment kits.

### Intervention

Treatment conditions:

1. Mexiletine hydrochloride 200 mg capsules, three times daily per os (PO)
2. Placebo capsules, three times daily PO

### Randomisation and preparation of study drugs

A statistician will generate computer-based randomization schemes, and will send these to the hospital pharmacy that is in charge of the distribution of medication. Mexiletine will be purchased from Agenzia Industrie Difesa - Stabilimento Chemico Farmaceutico Militare in Florence (Italy) where mexiletine is produced under Good Medicinal Practice (GMP) conditions. Mexiletine will be released by a Qualified Person.

Treatment kits will be prepared by the department of Pharmacy of our hospital. Following computer-generated randomization drug packaging and labelling will take place at our department of Pharmacy. At the start of the first set (see below) patients will receive a blinded, randomly-ordered treatment kit that contains medication for the entire set. In each set, patients will receive 4-week treatment with mexiletine, 200 mg 1 times a day PO (first week, day 1), 200 mg 2 times a day PO (first week, day 2) and 200 mg 3 times a day PO (remaining days of first week and second, third and fourth week), placebo will be provided in a similar build-up scheme with placebo tablets PO. The placebo is microcrystalline cellulose (supplied by Spruyt Hillen). The mexiletine and placebo are standard orange colored capsules (size nr. 00). We will count capsules to determine compliance at the end of the study. In case of study discontinuation, reasons will be documented.

### Outcomes and measurements

#### Primary outcome measure

##### Interactive voice response diary (IVR)

The primary endpoint will be the severity score of stiffness reported via the IVR. The primary endpoint is the same as in the RCT by Statland et al. [13]. The IVR is an automated centralized, real-time response phone-system that records severity and frequency of symptoms (stiffness, pain, weakness, and tiredness) and has been validated in patients with myotonia [30]. Patients will call in on a daily basis during the last two weeks of each four-weeks treatment period to rate (1) if they experienced symptoms during the past 24 hours and (2) the severity of the symptoms on an ordinal scale (1 to 9; 1 being minimal and 9 the worst ever experienced) using their telephone key pad. The IVR has been translated for this study [30].

#### Secondary outcome measures

Severity scores of pain, weakness, and tiredness will be documented as measured by the IVR from daily calls made by participants during the last two weeks of each four-weeks treatment period.

##### Questionnaires

1. The Individual Neuromuscular Quality of Life questionnaire (INQoL) has been validated in skeletal muscle channelopathies such as NDM [31]. The questionnaire consists of 45 questions within 10 sections, four sections focus on the impact of muscle disease symptoms, five sections focus on the impact of muscle disease on particular areas of life, and one section focuses on the positive and negative effects of treatment. A composite score can be derived representing overall QOL. No Dutch version was available. With granted authorization from the authors, a translation into Dutch language has been used in patients with a chronic neuromuscular disease [32].
2. The Short-Form 36-Item Health Status Survey (SF-36, Dutch version) is a generic questionnaire to establish the self-reported health status of patients. The questionnaire consists of the following domains: physical functioning (10 items), role functioning physical (four items), social functioning (two items), body pain (two items), mental health (five items), vitality (four items), general-health perception (five items), and change in health [33]. In addition summary composite scores for mental and physical functioning are derived. SF-36 domain scores from a Dutch nationwide sample of healthy individuals have been published [34].

##### Standardized interview

All interviews will be conducted by the same investigator (BCS). During the screening visit, answers to four open, standardized questions will be noted and recorded on video: 1. What is the most invalidating symptom or daily life disability that would make you take anti-myotonic medication? 2. Do you expect mexiletine to have a positive effect on your myotonic symptoms? 3. What kind of improvement would you need to experience for you to continue mexiletine treatment after the trial? 4. How important is a positive treatment-effect for you?

Apart from the standardized interview, co-morbidity, previous and current use of medication (also anti-myotonic treatment) will be reported. After each treatment set, the patients’ preference for one of the two treatment periods within the treatment-set, will be noted.

##### Clinical myotonia bedside tests

1. Eyelid closure action myotonia is defined as an increased muscle relaxation time of the orbicularis oculi and smaller upper and lower eyelid muscles (often between 1 – 15 sec) after forceful eyelid closure. The participant will be instructed to close the eyes as forcefully as possible for five seconds then rapidly open their eyes on command. This will be repeated five times in sequence. For each attempt, the time from the command to open their eyes until relaxation of the eyelid closure muscles will be timed and noted up to two decimals.
2. Hand-grip action myotonia is defined as an increased muscle relaxation time of the finger flexor muscles and some of the involved underarm flexor muscles (often between 1 – 15 sec) after a forceful handgrip. The participant will be instructed to forcefully close the fingers of the right hand in a fist (handgrip) for five seconds, while resting the back of the right hand on a table, and then, on command, rapidly open the fist on command. This will be repeated five times in sequence. For each attempt, the time from the command to open the right fist until relaxation of the handgrip muscles to the point that the fingertips of dig II-V reach the surface of the table will be timed and noted up to two decimals. Both eyelid-closure action myotonia and handgrip action myotonia have been previously used to redefine clinical phenotypes [8] or as therapeutic outcome measure [13].
3. The Timed Up&Go (TUG) measures the time in which the participant rises from a chair, walks three meters, turns around, walks back and sits down again in a self-selected speed. The TUG has shown intra- and inter-reliability and has been validated in patients with non-dystrophic myotonia [35,36].
4. Quantitative Grip Myotonia: Maximum Voluntary Isometric Contractions (MVICs) of the long finger flexors and the subsequent relaxation time (myotonia) will be measured using a technique developed at the University of Rochester [37]. To measure the extent of grip myotonia of resting forearm muscle, each participant will squeeze the grip handle with a maximum grip for three seconds then relax until the force returns to baseline. The relaxation time from 90% to 5% of maximal force following this initial MVIC will be used to calculate the degree of myotonia. Each participant will perform three sets of five MVICs. Each set will be separated by a 10-minute rest period. Additionally, for each MVIC, we will analyze the peak force (PF) and the decline (%) of this PF within the three seconds duration of the MVIC (as a measure for the phenomenon of transient paresis).

All clinical myotonia bedside tests will be conducted by the same examiner (BCS).

##### Measurement of myotonic discharges with needle-EMG

Concentric needle EMG will be performed in the left rectus femoris muscle at rest. This muscle was chosen based on our previous studies on NDM [8,38]. According to established criteria of Streib et al. [39] myotonic discharges will be defined and quantified during 10 insertions, each followed by 30 sec of visual and auditory identification of myotonic discharges. Myotonic discharges must be at least 500 milliseconds, with potential amplitudes ranging from 10 μV to 1 mV, waxing and waning in both amplitude and frequency. Grading of myotonic discharges: 0: No positive muscle activity or an occasional run of positive waves following needle movement (detection of myotonia in 0-2/10 insertions); 1+: Myotonia fulfilling the minimal requirements (detection of myotonic discharges in 3-5/10 insertions) 2+: myotonic discharges in more than one-half of needle insertions (detection of myotonic discharges in 6-9/10 insertions); 3+: myotonic discharges with each needle movement in all examined areas (detection of myotonic discharges in 10/10 insertions) [39]. The EMG signals will be amplified and filtered between 20 Hz and 3 kHz and stored using the liveplay feature of the Medelec Synergy EMG equipment (software version 10; Oxford Instruments Medical, UK) to facilitate future quantification of myotonic discharge characteristics [38]. All needle-EMG investigations will be performed by or under supervision of an experienced clinical neurophysiologist (CGS).

### Mexiletine-serum concentration

Serum samples for random drug levels determination will be obtained during the visits at the start and end of every treatment period. All serum samples will be analyzed at once, at the end of the trial, by our department of Pharmacology and Toxicology. Analysis will be based upon a previously published protocol using liquid chromatography [40].

### Cost-effectiveness

Health care consumption will be assessed by using a modified version of the Client Service Receipt Inventory (CSRI), which will be filled in by the patients at the end of each treatment period [41,42]. The CSRI measures direct neuromuscular disease-related health care costs (including costs of visits to other health care providers: GPs, specialist care, physical therapy, psychological therapy, social worker contacts), professional home care and hospitalization, as well as non-health care costs such as costs for paid and unpaid help. Additional prescribed and over the counter medication will also be recorded. For unit cost prices, standard rates will be adopted from the national guideline [43] or real cost prices (e.g., for medication) will be obtained through the website of the Dutch Health Care Insurance Boards (Zorginstituut Nederland, http://www.medicijnkosten.nl). The price year will be 2014 and the currency Euros. Costs per patient will be calculated by multiplying resource volumes by unit costs. Costs and effects (in terms of QoL) will be combined to assess cost-effectiveness. If mexiletine appears to be more effective and more expensive –or less effective and less expensive- than placebo, the cost-effectiveness will be expressed in terms of the incremental cost-effectiveness ratio (ICER). Nonparametric bootstrapping techniques will be used to produce confidence intervals around mean costs, mean effectiveness and (if necessary) the ICER.

### General structure of the study

Initial screening will be scheduled 2 weeks prior to the baseline visit. During study enrolment patients will have 4-16 outpatient clinic visits depending on number of treatment sets necessary to obtain enough evidence (Figure 1). Each visit will take approximately 1-1.5 hours and comprises two questionnaires, clinical myotonia bedside tests, ECG and venous blood collection for measurement of mexiletine blood serum levels. Needle-EMG investigations will be performed at the end of each treatment period (Table 2).

**Table 2:** **Schedule of study measurements during the screenings phase and the first treatment set**

		**Mexiletine or placebo** ***(period 1)***				**Wash-out and cross- over**	**Mexiletine or placebo** ***(period 2)***				**Interim analysis 1**	
**Actions**	**Screening**	**Week 1, day 1**	**Week 2, day 1**	**Week 3, day 1**	**Week 4, day 7**	**Week 5**	**Week 6, day 1**	**Week 7, day 1**	**Week 8, day 1**	**Week 9, day 7**	**Week 10**	**Week 11**
**Lab test**	X											
**Pregnancy test**	X											
**Consent**	X											
**Medical history**	X											
**ECG**	X				X					X		
**Needle EMG**					X					X		
**IVR**		X	X	X*	X*		X	X	X*	X*		
**INQoL/SF-36**		X			X		X			X		
**Clinical myotonia tests**		X			X		X			X		
**Quantative grip myotonia**		X			X		X			X		
**Mexiletine blood plasma levels**		X			X		X			X		
**Dispense drug/ placebo**		X										
**Collect medication bottles**					X					X		

Study measurements for a possible second, third and fourth treatment set are identical to the measurements in the first treatment set and are not represented in this table.

X* = daily collection of IVR data.

Patients who exhibit convincing evidence of a genuine positive effect will be offered to continue to use mexiletine (see section: statistical analysis – individual interim analyses). After completion of the individual N-of-1 trials, patients will be followed up for three months, to monitor clinical progression, drug compliance, adverse events and quality of life.

### Safety

ECGs taken at the beginning and end of each treatment period will be screened for abnormalities (conduction times: PR, QRS and QTc-time; and the presence of cardiac arrhythmia by an experienced cardiologist). Cardiac arrhythmias will be classified as clinically relevant or irrelevant. Clinically relevant arrhythmia and conduction disorders will be presented to the Data and Safety Monitoring Board (DSMB).

During the study, patients are instructed to directly report serious adverse events, non-serious adverse events (such as gastro-intestinal discomfort or nausea) are reported during trial visits.

### Quality assurance/monitoring

The Clinical Trial Center of the Radboud University Medical Center Nijmegen, Nijmegen (“Clinical Research Center Nijmegen” (CRCN); www.crcn.nl) will be responsible for the data quality management and monitoring of this trial.

### Ethical approval and registration

This study has been reviewed and approved by the medical ethics committee of the region Arnhem-Nijmegen, The Netherlands, (reference CMO nr. NL34801.091.10) and has been registered at clinicaltrials.gov (ClinicalTrials.gov Identifier: NCT02045667). Patients receive verbal and written information about the study and written informed consent will be obtained before randomization.

### Data safety monitoring board

There will be bi-annual controls by the data safety monitoring board, which consist of a clinical pharmacologist, a cardiologist and a biostatistician. This committee will analyze all severe adverse events, drop-outs due to adverse medical events and mortalities.

### Statistical analysis

#### Sample size and power

The IVR measure for stiffness (a 9-points scale) is our primary endpoint. No minimal clinically important difference (MCID) calculation for the IVR was available from the literature. We chose an MCID of 0.75 on the mean IVR-score as clinically relevant difference based on clinical expert opinion and a previously reported within subject standard deviation (SD) of 1.5 (with ½ SD as a distribution-based estimate for the MCID [44]) [30]. The mean IVR score in the published RCT was 4.21 (95% confidence interval 3.40 - 5.20) on placebo [13]).

The data of the combined N-of-1 trials will be analyzed using a hierarchical Bayesian model [45]. Since no formula-based methodology exists for sample size or power calculations for such designs, we have performed a simulation-based sample size calculation.

The simulation-based sample size calculation consisted of the following steps: (1) Drawing a random realization from a prior distribution for the mean treatment effect, based on expert opinion. (2) For each of these realizations, we have simulated data for 30 N-of-1 trials using R software, where the data for each N-of-1 trial consisted of 2 × 10 × 2 observations (for 2 treatment arms, 10 observations per arm per treatment pair, and 2 completed treatment pairs). These simulations were performed using a model structure with a random intercept and a random slope for different individuals and a residual within-person error. (3) Each simulated data set of 30 N-of-1 trials was analyzed as described by Zucker et al. [22]. We have combined each of the simulated data sets with a normally distributed prior distribution with a mean of 1.75 and a standard deviation of 0.89, which was also based on expert opinion. (4) Each of these Bayesian analyses resulted in a marginal posterior distribution for the mean treatment effect β_0_. (5) For each of these posterior distributions we determined the posterior probability of a treatment effect of at least 0.75. We assumed that all participants will be subjected to two treatment pairs. Steps 1 - 5 were re-iterated 1000 times, resulting in 1000 posterior probabilities of a clinically meaningful treatment effect. From these 1000 probabilities we have determined the mean, which corresponds to an estimate of the expected posterior probability of a substantial treatment effect. The mean expected posterior probability of a clinically meaningful treatment effect was 0.82. Thus, under the specified assumptions, with the results of 30 patients with NDM completing the trial, we would be 82% certain that mexiletine produces a clinically meaningful treatment effect in these patients.

#### Prior elicitation, informative and non-informative priors

The strength of Bayesian analyses is that it provides an algorithm for updating the probability estimate of a particular claim being true (e.g., the claim that mexiletine produces, on average, an improvement of at least 0.75 on the IVR stiffness scale in patients with NDM, when compared to placebo) whenever novel relevant evidence becomes available. The information that was already available before the novel evidence became available is expressed as prior distributions (so called “priors”). For all model parameters a prior will be needed, however, since the mean treatment difference is the parameter of main interest, these priors will receive the most attention. In the main analysis an informative prior based on data on the treatment effect from the previous study (i.e. treatment effect in the RCT by Statland et al. [13]) will be used. In a sensitivity analysis we will use a prior for the treatment effect based on expert opinion, in which case they are elicited from expert physicians: expert neuromuscular neurologists with experience in the pharmacological treatment of NDM patients. These neurologists will be asked to estimate the treatment effect based on patient demographic information, genetic information and video-clips of the baseline myotonia bedside tests. A histogram-based method (Spiegelhalter ([46], p.145) will be used to elicit individual (i.e. from each neurologist) clinical priors, which will subsequently be aggregated to provide one group clinical prior. Another sensitivity-analysis will be performed with a non-informative prior for the group level mean treatment effect instead of the informative priors. For all other model parameters we will use non-informative (or ‘flat’) priors, since it is difficult to elicit parameters such as random effect variations from physicians [46].

#### Individual interim analyses

After treatment sets 1, 2 and 3 of each N-of-1 trial we will investigate whether the existing evidence at that moment is sufficient to conclude that one of the two treatments is more effective for that particular individual. This will be done by a statistician who is blinded for treatment allocation and who will use Bayesian methods. The patient and the treating physician will be advised to discontinue the N-of-1 trial if the posterior probability of a treatment effect larger than 0.75 (clinically relevant mean difference) is at least 80% (discontinue trial participation and start regular treatment) or at most 20% (discontinue trial participation and do not start regular treatment). In all other cases they will be advised to continue the N-of-1 trial. Taking into account this advice, the physician and patient will discuss the effects of treatment, as observed by the physician and experienced by the patient, and together they will decide whether or not to continue the N-of-1 trial.

For our interim analysis we will use non-informative priors only, as we prefer to base our stopping advice on the patient data.

#### Bayesian hierarchical analysis

This study aims to answer the following questions: What is the probability that mexiletine is clinically effective in patients with non-dystrophic myotonia (NDM) on the individual and group level? To combine the results of the multiple N-of-1 trials, a hierarchical (multi-level) Bayesian model will be used, with the IVR measure for stiffness as the dependent variable, and with the patient and the subgroup (chloride versus sodium channel mutation carriers) as the structural grouping factors (or the levels of the model). The patient will be treated as a random effect (both a random intercept and a random slope), while subgroup of patients and mutation type will be treated as fixed effects. The within person residual variance will similarly be assumed to be drawn from a common distribution. In the main analysis, the prior will be based on the RCT results from Statland et al [13]. Sensitivity analyses with a clinical prior and with a non-informative prior will also be performed. From the Bayesian analysis we will obtain posterior distributions for the mean treatment effect at the population level, as well as posterior distributions for the treatment effects at the individual level, that will exhibit borrowed strength from the population estimates through shrinkage to the population mean. For details of the procedure to be used, see Zucker et al. [22].

Also, we will investigate interactions between treatment effect and treatment set, and between treatment effect and treatment order. Secondary endpoints will be analyzed similarly.

#### Comparisons with the RCT and traditional analysis methods

In addition to synthesizing the data from our study with the existing RCT evidence, we will also contrast the outcomes of the two studies. To this end, a sensitivity analysis with a non-informative prior will be performed. This will provide a direct comparison of the outcomes of the two studies. In order to compare the novel methodology of combined N-of-1 trials with more traditional analysis methods, we will perform a (non-Bayesian) frequentist analysis of our data, where the same approach will be chosen as in the previous cross-over RCT by Statland et al. [13].

## Discussion

In conclusion, our study offers a unique opportunity to assess the validity and feasibility of Bayesian analyses of combined N-of-1 trial methodology to obtain evidence of the clinical and cost-effectiveness of drugs for rare diseases, at the individual and group level simultaneously.

Furthermore, our approach, if valid and feasible, may reduce costs compared to an international RCT, and enables clinicians to potentially run trials in the setting of out-patient clinic visits.

As such, our study may serve as a model for future research into treatments in other rare genetic diseases, and will help to bridge the gap between research and clinical practice.

Apart from this methodological objective, results of our study will contribute to the current level of evidence of the clinical-effectiveness of mexiletine in NDM and may clarify the cost-effectiveness of mexiletine treatment in NDM patients.

## Abbreviations

NDM: Non-dystrophic myotonia
NDMs: Non-dystrophic myotonic syndromes
CINCH: Consortium of clinical investigation of neurological Channelopathies
CVZ: National health insurance board of The Netherlands
RCT: Randomized controlled trial
EMA: European medical agency
FDA: Food and drug administration
EU: European union
CLCN1: Skeletal muscle chloride channel gene
SCN4A: Skeletal muscle sodium channel gene
PO: Per Os
BMC: Becker myotonia congenita
TMC: Thomsen myotonia congenita
PMC: Paramyotonia congenita
SCM: Sodium channel myotonia
IVR: Interactive voice response system
EMG: Electromyogram
ECG: Electrocardiogram
INQoL: The Individual neuromuscular quality of life questionnaire
SF-36: The Short-form 36-item health status survey
TUG: The Timed up & go
MVIC: Maximal voluntary isometric contraction
PF: Peak force
ICER: The incremental cost-effectiveness ratio
CSRI: The client service receipt inventory
CRCN: Clinical research centre nijmegen
DSMB: Data and safety monitoring board
ZonMw: The Netherlands organisation for health research and development
QoL: Quality of life
GP: General practitioner
MCID: Minimal clinically important difference
